# Supplementary material for: EnzML: multi-label prediction of enzyme classes using InterPro signatures
Source: BMC Bioinformatics. 2012 Apr 25;13:61. doi: 10.1186/1471-2105-13-61 (PMC3483700; doi:10.1186/1471-2105-13-61)
Supplement: Addtional file 5 — The Java code to format the data files, evaluate and predict. The file enzml_java_code.tar.gz contains the Java code used to format database data to ARFF and XML formats, to execute cross and train-test (jackknife) evaluations and to record evaluation results to database. More information is included in the readme.txt file and the Javadoc files. The code can be used with a MySQL database. To use a different database software, other JDBC drivers might be required. [file 1471-2105-13-61-S5.gz › java_code/enzml2011/doc/uk/ac/ed/inf/taweka/mulan/learn/class-use/MulanLearner.html]

Uses of Class uk.ac.ed.inf.taweka.mulan.learn.MulanLearner


---


|  |  |  |
| --- | --- | --- |
| **Overview** \| **Package** \| **Class** \| **Use** \| **Tree** \| **Deprecated** \| **Index** \| **Help** | |  |
| PREV   NEXT | **FRAMES**    **NO FRAMES**     **All Classes** |  |


---


## **Uses of Class uk.ac.ed.inf.taweka.mulan.learn.MulanLearner**

| Packages that use MulanLearner | |
| --- | --- |
| **uk.ac.ed.inf.taweka.mulan.learn** |  |
| **uk.ac.ed.inf.taweka.test.mulan.learn** |  |

Uses of MulanLearner in uk.ac.ed.inf.taweka.mulan.learn

| Methods in uk.ac.ed.inf.taweka.mulan.learn that return MulanLearner | |
| --- | --- |
| `MulanLearner` | `MulanSerializer.getLearner()` |
| `MulanLearner` | `MulanCrossExperimenter.getLearner()`             public String getExperimentPropsPath() { return m\_experimentPropsFile; } |
| `MulanLearner` | `MulanCrossEvaluator.getLearner()` |

| Methods in uk.ac.ed.inf.taweka.mulan.learn that return types with arguments of type MulanLearner | |
| --- | --- |
| `static java.util.Vector<MulanLearner>` | `MulanLearners.getLearners()` |

| Methods in uk.ac.ed.inf.taweka.mulan.learn with parameters of type MulanLearner | |
| --- | --- |
| `static uk.ac.ed.inf.utils.database.TableRow` | `ResultsFormatter.createEvaluationRecord(int runNumber, int trainArffId, int testArffId, java.lang.String startTime, java.lang.String endTime, MulanLearner learner, mulan.evaluation.Evaluation evaluationResults, uk.ac.ed.inf.utils.database.TableManager tableManager)`             Add timestamps and identifiers to the cross evaluation results |
| `int` | `ResultsSaver.saveResults(int runNumber, int trainArffId, int testArffId, java.lang.String startTime, java.lang.String endTime, MulanLearner learner, mulan.evaluation.Evaluation evaluationResults)` |

Constructors in uk.ac.ed.inf.taweka.mulan.learn with parameters of type MulanLearner

`MulanCrossExperimenter(java.lang.String resultsDbPropsFile,
java.lang.String arffRecordDbProps,
int trainArffRecordId,
MulanLearner learner)`

`MulanSerializer(MulanArffRecord arffRecord,
MulanLearner learner,
java.lang.String directoryForSerializedModel)`

`TrainTestExperimenter(java.lang.String experimentPropsFile,
java.lang.String arffRecordDbProps,
int trainArffRecordId,
int testArffRecordId,
MulanLearner learner)`

`TrainTestExperimenterSerialized(java.lang.String experimentPropsFile,
java.lang.String arffRecordDb,
int trainArffRecordId,
int testArffRecordId,
MulanLearner learner,
java.lang.String trainedModelFile)`

Uses of MulanLearner in uk.ac.ed.inf.taweka.test.mulan.learn

| Methods in uk.ac.ed.inf.taweka.test.mulan.learn that return MulanLearner | |
| --- | --- |
| `static MulanLearner` | `LearnerTest.getMulanLearnerMLkNN()` |
| `static MulanLearner` | `LearnerTest.getMulanLearnerOneR()` |
| `static MulanLearner` | `LearnerTest.getMulanLearnerZeroR()` |

---


|  |  |  |
| --- | --- | --- |
| **Overview** \| **Package** \| **Class** \| **Use** \| **Tree** \| **Deprecated** \| **Index** \| **Help** | |  |
| PREV   NEXT | **FRAMES**    **NO FRAMES**     **All Classes** |  |


---
